# Supplementary material for: Topical Application of Phlorotannins from Brown Seaweed Mitigates Radiation Dermatitis in a Mouse Model
Source: Mar Drugs. 2020 Jul 22;18(8):377. doi: 10.3390/md18080377 (PMC7460453; doi:10.3390/md18080377)
Supplement: Supplementary file 1 [file marinedrugs-18-00377-s001.pdf]

marine drugs

SUPPROTING INFORMATION

Topical application of phlorotannins from brown seaweed mitigates radiation dermatitis in a mouse model

Kyungmi Yang [1,2,†], Shin-Yeong Kim [1,†], Ji-Hye Park [2,3], Won-Gyun Ahn [1], Sang Hoon Jung [1], Dongruyl Oh [1,3], Hee Chul Park [1,3,*] and Changhoon Choi [1,*]

[1] Department of Radiation Oncology, Samsung Medical Center, Seoul 06351, Republic of Korea; kyungmi.yang@samsung.com (K.Y.); kkdnsy@naver.com (S.-Y.K.); mementoamor@icloud.com (W.-G.A.); sang-hoon.jung@samsung.com (S.H.J.); dongryul.oh@samsung.com (D.O.)
[2] School of Medicine, Sungkyunkwan University, Seoul 06351, Republic of Korea; jh1024.park@samsung.com (J.-H.P.)
[3] Department of Dermatology, Samsung Medical Center, Seoul 06351, Republic of Korea
† These authors contributed equally to this work.
* Correspondence: hee.ro.park@samsung.com (H.C.P.); chchoi93@gmail.com (C.C.); Tel.: +82-2-3110-2605 (H.C.P.); +82-6190-5331 (C.C.)

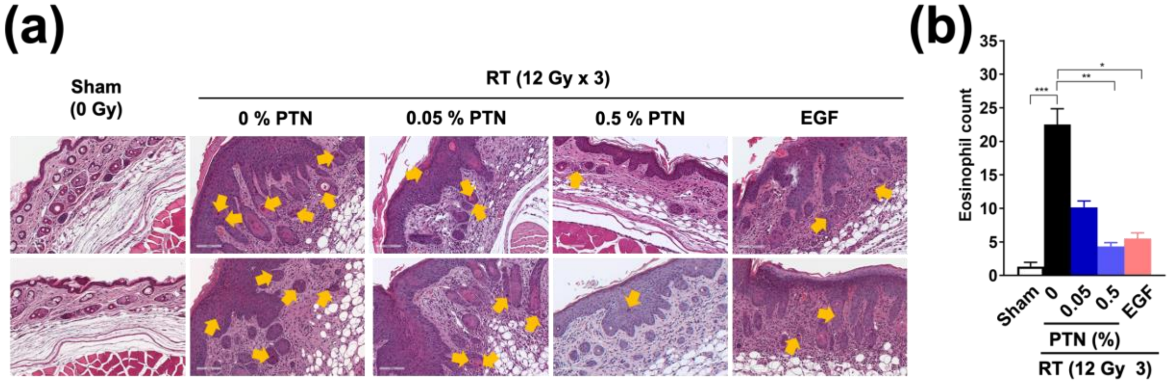

Figure 1. Effects of phlorotannins on infiltration of eosinophils in irradiated skin tissues. **(a)** Representative two images of H&E staining of irradiated skin tissues. Skin are was topically treated with 0%, 0.05%, and 0.5% PTNs or EGF. Skin tissues were collected at 14 days post-irradiation. Yellow arrows indicate eosinophils. **(b)** Quantification of eosinophils infiltrating in irradiate skin tissues. Data are means ± SD (n = 6 per group); Difference was evaluated using a Kruskal-Wallis test, followed by Dunn's multiple comparison test. * p <0.05; ** p <0.01; *** p < 0.001.

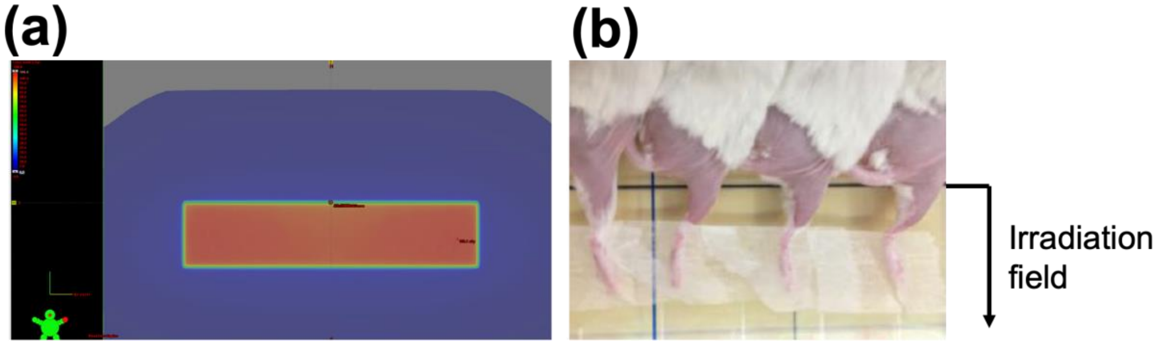

Figure 2. Setup for mouse irradiation. **(a)** A treatment plan showing 30 cm × 7 cm field size. **(b)**. A photo image showing that right hind legs were located within the irradiation field.
